# Intelligent Cloud Resource Usage Potentially to Improve Task Scheduling by the use of Artificial Intelligence

**DOI:** 10.12688/f1000research.177203.2

**Published:** 2026-06-25

**Authors:** Huda Mohammed Lateef, Mohammed Abduljawad Al-Shibly, Ahmed Hadi Ali AL-Jumaili, Omar D. Madeeh, Mohammed A.S. Al-Hitawi, Deshinta Arrova Dewi, Mohd Zakree bin Ahmad Nazri, Shaima Nasir Kadhim

**Affiliations:** 1 University of Fallujah, Al-Fallujah, Al Anbar Governorate, Iraq; 2 INTI International University & Colleges, Nilai, Negeri Sembilan, Malaysia; 3 Faculty of Data Science and Information Technology, Universiti Kebangsaan Malaysia, Bangi, Selangor, Malaysia; 4 University of Diyala, Baqubah, Diyala Governorate, Iraq

**Keywords:** Cloud Resource Utilization, Scheduling, Resource Management, Machine Learning (ML), Deep Learning, Google Cluster.

## Abstract

**Background:**

The high variability of workloads makes it very difficult for cloud datacenters to efficiently schedule their tasks and resource allocation. The correct forecasting of future resource utilization allows us to proactively scale and implement more sophisticated scheduling policies, which eventually results in has potential to improve resource utilization and fewer failures.

**Methods:**

This study uses the Google Cluster Trace v3 dataset, which contains a wealth of job and task data (start and end times, CPU and memory usage, scheduling class, and priority) to create supervised machine and deep-learning models that can predict future of CPU usage.

**Conclusions:**

This study evaluates Linear Regression (LR), Support Vector Regression (SVR), Random Forest (RF), and Neural Network (NN) models for predicting future CPU usage, which are quite successful in predicting computer resource usage (94% validation accuracy and R ^2^ of 0.90). These results underscore the potential of data driven approach definitely machine and deep learning-based CPU demand predictions as valuable tools for cloud schedulers, improving resource management, and reducing operational costs. Also discuss the feasibility of deploying the proposed solution on distributed platforms such as Spark and Google Cloud and outline future research directions to integrate predictive models with real-time cloud resource management.

## 1. Introduction

Cloud computing has become a basic infrastructure for modern information technology services that provide on-demand access to scale-based computational services and it is part of our everyday for our life. The essential properties of elasticity, resource pooling, and measurable services of cloud computing were outlined in a seminal exposition by Armbrust et al. (2010). In modern cloud data centers, resource and task scheduling are essential for ensuring performance and cost-effectiveness. The dynamic quality of cloud workloads, which is inherently defined by the presence of tasks with dissimilar and stochastic CPU and memory demands, makes it suboptimal to employ static or rule-based scheduling frameworks. Therefore, predictive resource allocation has become an attractive paradigm to alleviate this predicament. Smart cloud systems can predict the future utilization of resources using artificial intelligence (AI) and machine learning (ML) in addition to deep learning (DL) technologies, which can be used to proactively modify scheduling policies to prevent over-provisioning and bottlenecks, thus improving overall operational effectiveness, to save energy and contributing to achieving the Sustainable Development Goals (SDGs). Experimental research studies have revealed that the intersection of AI and big data analytics in cloud operations is a significant addition to forecasting accuracy and operational effectiveness.

The Borg system is a large-scale cluster manager on Google, which is an example of a complex scheduler that coordinates thousands of jobs on many machines. Even though such systems use sophisticated heuristics, machine learning predictions can still be used to further optimize the scheduling performance. In 2019, Google published the Google Cluster Trace (v3) dataset, which provides a complete dataset of real-world cloud workload data to be studied. The trace contains approximately 2.4TiB of data sampled between eight datacenter clusters over a period of one month, including detailed entries of job identifiers, task identifiers, submission and completion times, resource usage measures (CPU, memory), priority, scheduling classes, and other auxiliary information. The results of the analysis of this dataset show that the variability of task phenomenology and resource consumption is strong, and that the correlations between the attributes, including priority or constraints, and the scheduling outcomes can be identified. Workloads, which are heterogeneous (some tasks are short and low-priority, while others are long-lasting and high-priority services) pose insurmountable obstacles to homogeneous scheduling policies. In this regard, the deployment of learning-based predictive models, which can identify intricate patterns hidden in historical traces and forecast future resource needs of incoming tasks, is highly encouraged.

In this paper, we describe a thorough exploration of the prediction of CPU utilization of cloud tasks using supervised machine-learning schemes. Our most important goal is to improve cloud resource management by allowing predictive task scheduling by predicting the future CPU demand of a task, thus allowing the scheduler to schedule resources or make placement decisions. We compare a collection of regression algorithms, such as linear regression, support-vector regression, random forests, gradient-boosting machines, and custom neural networks, and suggest hybrid ensemble approaches to improve predictive accuracy. Two specific hybrid methods are discussed: (1) a voting ensemble, which combines the predictions of several constituent models, and (2) a clustering-based ensemble, which first divides tasks with similar properties and then builds specialized predictors on each cluster. In contrast to these approaches, we attempted to find the most correct and generalizable approach to this predictive undertaking.

The contributions of this research include providing an in-depth evaluation of traditional and modern machine-learning models on a large-scale cloud trace dataset to the CPU prediction problem. In addition, we performed a detailed examination of empirical results such as model performance statistics Mean Absolute Error (MAE) and Root Mean Squared Error (RMSE) and training behavior to explain the stability and generalization properties of the learned models. The best model achieves a large predictive accuracy (with R ^2^ near 0.90 and very low error rates), a result that, as far as we can discern, is comparable to or better than most existing models applied to similar data. We also address real-world deployment scenarios, such as the use of distributed systems such as Hadoop/Spark or cloud-native services, to support real-time scheduling in clusters of production. All experiments were publicly available at the AlgoEval-GCD repo ^1^ and were reproducible.

## 2. Related work

The scheduling of cloud computing resources continues to be a persistent research topic, particularly owing to the dynamic characteristics of cloud workloads ( Kareem Awad et al., 2025). Conventional approaches, mainly heuristic or rule-based algorithms, find it challenging to accommodate the extensive scale and variety of workloads. Recent breakthroughs in artificial intelligence and machine learning have facilitated the development of more adaptive and efficient systems, enabling the prediction of future resource demands and dynamic allocation of resources. AI-driven methodologies, including reinforcement learning (RL), offer a framework for autonomously identifying optimal scheduling strategies through interactions with cloud environments and data-driven learning.

Numerous pivotal studies have investigated the use of machine learning in resource allocation and workload forecasting. This section examines these studies, focusing on their contributions to job scheduling, resource allocation, and the incorporation of AI in cloud environments, specifically within the Google Cluster design framework.

### 2.1 Analysis of production trace data for scheduling optimization

Zarour et al. (2024) conducted an early study analyzing the Google Cluster Trace dataset to examine the influence of task variables such as CPU and memory needs, scheduling priority, and task limitations on job scheduling efficiency. Their findings indicated that specific criteria, such as memory availability, significantly influenced execution time and rescheduling frequency, whereas others, such as stringent job requirements, had a diminished impact. This study did not offer an AI-based solution; instead, it established a basis for AI-driven scheduling models by identifying essential scheduling parameters to be integrated into machine learning models. This study substantially advanced feature engineering for cloud scheduling systems by identifying the most influential aspects affecting task performance.

### 2.2 Machine learning models for workload prediction and resource allocation

Gao et al. (2020) proposed a workload prediction system that categorizes workloads prior to employing a singular machine learning model for each category. Their research utilizing Google Cluster data revealed that categorizing clustering jobs by their attributes resulted in markedly enhanced prediction accuracy (approximately 90%) compared to individual models. This discovery underscores the advantage of considering workload homogeneity through the aggregation of analogous jobs, enabling models to specialize in correctly predicting specific workload categories.

Karpagam and Kanniappan (2025) introduced an innovative model for forecasting cloud resource time series using a symmetry-aware multidimensional attention spiking neural network (SAMS-SNN). Spiking Neural Networks (SNNs) are recognized for their capacity to efficiently process temporal data while utilizing minimum energy, rendering them appropriate for time-series forecasting in cloud settings, were they known as “Fire together and Wire together”. The research employed attention processes to emphasize significant traits and utilized optimization methods, such as Secretary Bird Optimization (SBOA), to improve prediction accuracy. This approach substantially surpasses conventional recurrent neural networks (RNNs), such as Long Short-Term Memory (LSTM) and Gated Recurrent Units (GRU), in terms of efficiency and precision.

Although our methodology does not utilize spiking neural networks, it is influenced by the research of Karpagam et al., specifically with the application of attention processes to emphasize the most pertinent aspects. Similar to Karpagam et al., we investigated the efficacy of deep learning models, including feed-forward networks, for forecasting CPU loads in cloud environments, illustrating that tailored deep learning models can enhance prediction accuracy compared to conventional methods.

### 2.3 Predicting failures and resource usage in cloud environments

Chen et al. (2014) investigated the failure patterns of tasks within Google clusters, emphasizing the capability of predictive models to proactively identify errors. Their emphasis on failure prediction highlighted the significance of proactive forecasting in cloud operations. Anticipating peak demand or possible resource utilization issues enables cloud systems to implement remedial measures such as job rescheduling or additional resource supply prior to the emergence of problems.

Mishra et al. (2010) examined resource usage variability and task inter-arrival time in Google Cloud backends. Scientists have found that cloud workloads exhibit a great deal of diversity, which is problematic in terms of resource allocation and scheduling. Predictive models should have the ability to generalize between different workloads to eliminate this unpredictability.

### 2.4 Hybrid approaches and combining domain knowledge with AI models

The existing literature highlights several important results on how to optimize the process of cloud resource scheduling and workload prediction, among which the nature of tasks such as task priority and resource requests plays an important role in determining the scheduling results. Clustering-based models, deep neural networks, and reinforcement learning methods of machine learning have shown promise for improving the management of cloud resources ( Al-Jumaili et al., 2026).

These insights were synthesized in our research to create an ensemble methodology in which a large number of models (e.g., Random Forest (RF), Gradient Boosting (GB), and predicted Neural Networks (NNs)) are combined to increase the precision of the predictions. Moreover, our investigations will be based on previous research by providing an in-depth analysis of the model training dynamics and generalization properties, which ensures that our models are accurate and stable under different workload conditions.

In multiple cloud services conditions, Sefati et al. (2025) suggested a versatile resource scheduling methodology combining metaheuristic optimization and recurrent neural forecast. In accordance to their analysis, predictive scheduling may minimize delays and service-level obligation breaches while raising the utilization of resources.

Table 1 highlights exemplary studies regarding scheduling strategies, failure prediction, and cloud burden analysis, emphasising the emergence of predictive modelling techniques as well as the contributions they have made.

**Table 1.** Summary table of related work.

Study	Focus	Contribution
Mishra et al. (2010)	Resource usage and task inter-arrival times in Google clusters	Explores the heterogeneity of cloud workloads and the need for generalizable predictive models to handle various workload regimes.
Chen et al. (2014)	Predicting failures in cloud clusters	Identifies the potential of predictive models for early failure detection in cloud clusters, supporting proactive resource management.
Gao et al. (2020)	Workload prediction with task clustering	Demonstrates the benefits of clustering tasks based on workload characteristics to improve prediction accuracy (90% accuracy).
Li et al. (2021)	Dynamic job scheduling using deep reinforcement learning (DRL)	Proposes an RL model that learns optimal scheduling policies through interaction with a cloud environment.
Zarour et al. (2024)	Impact of task parameters on scheduling efficiency	Provides insights into task characteristics (e.g., CPU, memory) that influence scheduling efficiency and rescheduling needs.
Karpagam and Kanniappan (2025)	Workload and resource time-series prediction using SNN	Introduces a Symmetry-Aware Multi-Dimensional Attention Spiking Neural Network (SAMDA-SNN) to predict workload time series with high accuracy.

Yao et al. (2025) used multi-agent reinforcement learning in cloud-native systems for resource orchestration. The framework uses different agents for various system components and combines local and global feedback during training. Their results showed better stability than traditional approaches especialy for resource utilization, scheduling performance, and overall system.

In summary, the union of AI and machine learning in the sphere of scheduling cloud resources and predicting workload is a rapidly growing field. We based our work on previous research that combined clustering methods, deep learning types, and the ensemble strategy to enhance the accuracy and stability of the estates. This study expands the existing capacities of cloud task scheduling and helps create a more efficient cloud resource management system based on AI.

## 3. Dataset description

This study employed the publicly accessible Google Cluster Trace v3 dataset, which comprises extensive logs from Google’s computer clusters. The dataset collected in May 2019 spans millions of task instances executed across thousands of machines. Each record in the dataset included several important features: Job ID and Task ID (identifiers used to group tasks into jobs), timestamps (task submission/start and end times), resource usage metrics (CPU and memory usage throughout a task’s lifetime), scheduling class (indicating the task’s priority/latency sensitivity), and priority level (an integer assigned by the scheduler). As we can see in Figure 1 a small number of outliers in the dataset were removed so the most of continues distributions are uniforms.

**Figure 1.**
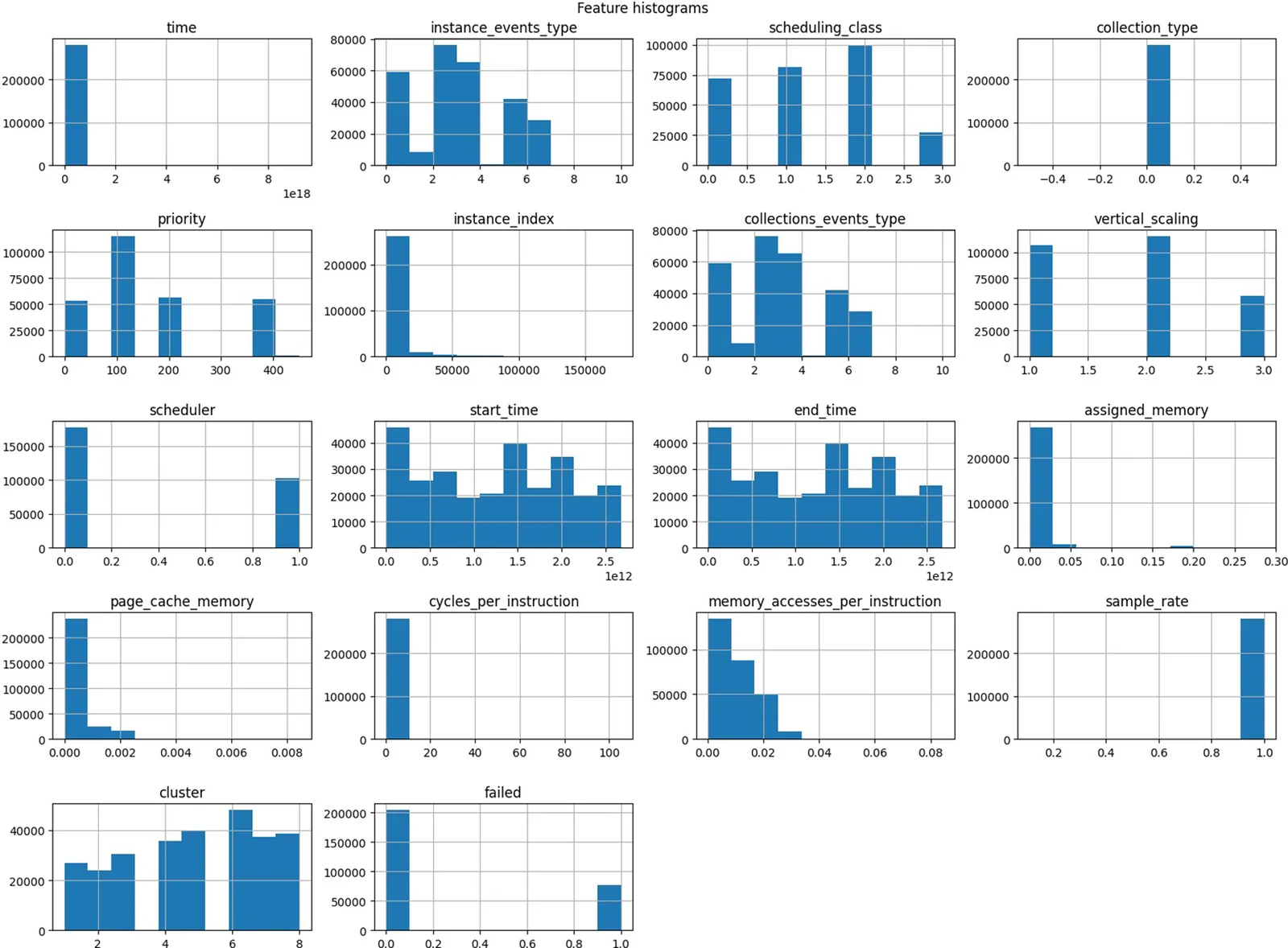
Distribution of the dataset after outlier removal.

Table 2 shows statistical description for the utilized google cluster benchmark, foreach feature we have short description and how many of samples was used in this study. Mean and standard division and mini max scale measurement in addition to the median.

**Table 2.** Statistical description for the used dataset.

Feature	Description	Count	Mean (± Std)	Min – Max	Median (IQR)
time	Event timestamp (ns)	405,894	6.9e13 ± 2.5e16	0 – 9.2e18	1.08e12 (2.67e11–1.77e12)
instance_ events_type	Type of task event (0–10)	405,894	2.95 ± 2.04	0 – 10	3 (2–5)
collection_id	Job/collection identifier	405,894	3.6e11 ± 2.4e11	6.8e3 – 8.2e11	3.1e11 (2.1e11–4.9e11)
scheduling_class	Scheduling class (0–3)	405,894	1.27 ± 1.01	0 – 3	1 (0–2)
priority	Scheduling priority (0–450)	405,894	147.9 ± 116.7	0 – 450	105 (103–200)
machine_id	Machine identifier (unique, can be -1)	405,894	8.5e10 ± 1.38e11	-1 – 8.25e11	2.0e10 (2.9e9–1.3e11)
resource_ request_cpus	Requested CPU cores	405,894	0.015 ± 0.029	0 – 0.58	0.008 (0.004–0.016)
resource_ request_memory	Requested memory fraction	405,894	0.009 ± 0.022	0 – 0.29	0.003 (0.001–0.007)
average_ usage_cpus	Average CPU usage fraction	405,894	0.007 ± 0.019	0 – 0.54	0.001 (0.0002–0.007)
average_ usage_memory	Average memory usage fraction	405,894	0.0056 ± 0.017	0 – 0.22	0.001 (0.0002–0.004)
failed	Failure flag (0 = no fail, 1 = fail)	405,894	0.23 ± 0.42	0 – 1	0 (0–0)

Figure 2 shows the Correlation Matrix ( *Cor*( *x,y*)), which represents the strength of the relationship between viables.

**Figure 2.**
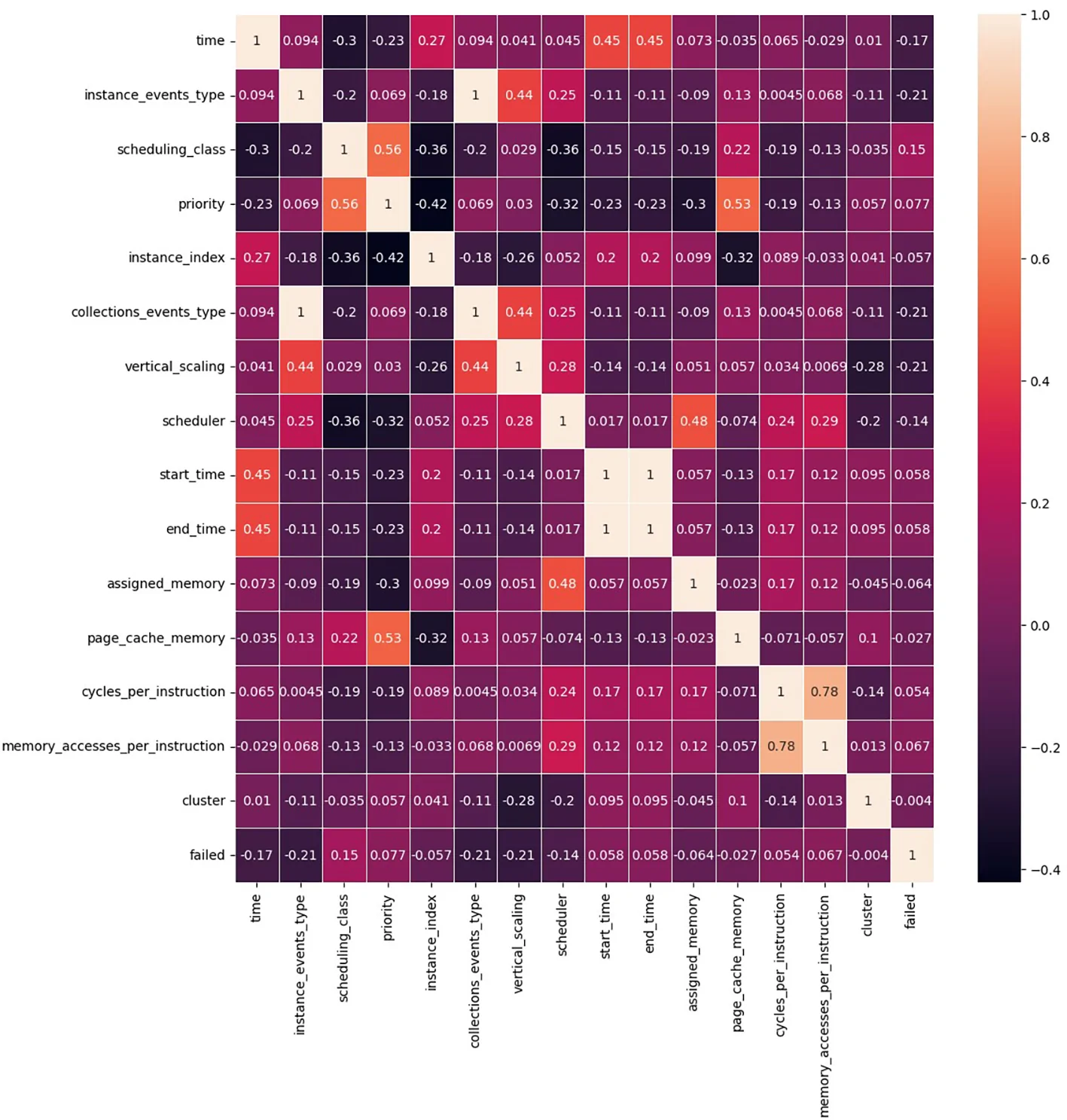
Show the correlation matrix for the used dataset.

The Equation (1) represents the analysis of the correlation matrix, the prime above the *X* and *Y* variables represents the mean values, *s _x_* and *s _y_* are the standard divisions of the given variables.

$$\mathrm{Cor}\left(X,Y\right)=\frac{\mathrm{Cov}\left(X,Y\right)}{s_{x}*s_{y}}=\frac{\sum \left(x_{i}-\bar{x}\right)*\left(y_{i}-\bar{y}\right)}{\sqrt{\sum {\left(x_{i}-\bar{x}\right)}^{2}*{\left(y_{i}-\bar{y}\right)}^{2}}}$$ (1)

The value is strongly related when it is close to ***1*** or ***-1*** and weak when it is zero or near it.

## 4. Methodology

### 4.1 Preprocessing

For this study, we utilized the publicly available Google Cluster Trace v3 dataset, which contains comprehensive logs from Google’s computer clusters.

$$x_{\text{scaled}}=\frac{x-x_{\min}}{x_{\max}-x_{\min}}$$ (2)

The target variable of our study is future CPU usage, specifically, the maximum CPU consumption during a specific future window (e.g., the upcoming scheduling interval). Our goal is to predict future CPU demand based on the information available at the time of task dispatch.

### 4.2 Feature engineering

The feature engineering process involved extracting relevant attributes that are available at the time of task scheduling, which included the requested CPU and the amount of CPU the task requested during submission. Amount of memory requested for the task during submission.

Class Scheduling is a categorical feature that classifies tasks according to their priority and latency sensitivity. The priority level represents a numeric feature assigned to a task by the scheduler. In addition, the duration estimates the runtime of the task, which can be derived from metadata or calculated by subtracting the start time of the task from its end time.

The CPU load on the host machine at the time of scheduling, assuming task placement, could account for the current host activity. To facilitate model training and ensure convergence, continuous features such as CPU, memory, and duration were scaled to a range of [0,1] using min-max normalization. Categorical features, such as the scheduling class and priority level, were encoded using one-hot encoding.

Defining the Target Variable

The regression model dependent variable was the proportion of predicting the future CPU demand using only scheduling-time features by a job during its execution. This was computed by dividing the CPU usage by the time that the task was being executed, giving a value in the range of 0-1. A value of 1 indicates the full usage of one of the CPU cores, and a value near 0 indicates less work. In a situation where jobs were forcibly displaced or cancelled, the CPU consumption at the time of cancellation was used.

We also performed binary classification with a continuous regression model to determine high- and low-load jobs (i.e., those that used more than 50 percent of a CPU core) by defining a threshold of 50 percent CPU usage. The given classification method provides real advantages to the person scheduling jobs by identifying the jobs that might require a specific management approach, including dedicated CPU utilization or higher priority in resource allocation.

Runtime metrics were excluded to avoid data leakage, only scheduling-time attributes were used for modeling.

Data Volume and Sampling

Owing to the extensive size of the dataset, which includes millions of tasks, training machine learning models on the complete dataset is computationally prohibitive. Consequently, we used a sampling approach to establish a feasible subset for our tests. We randomly selected a heterogeneous selection of jobs encompassing multiple scheduling classes and priority levels and incorporated all tasks related to those jobs. This sample comprised hundreds of thousands of task instances, sufficiently large to train robust models, yet compact enough to fit into memory for efficient processing.

The dataset was divided into three subsets: 70 percent of the data were utilized as the training set for model development, 15 percent were assigned as the validation set for hyperparameter optimization and cross-validation, and the remaining 15 percent were used as the test set to assess the final model performance. The temporal division guaranteed that the validation and test sets comprised tasks from subsequent time intervals, mirroring the actual context of forecasting future unobserved workloads.

Dealing with Imbalanced Data

We also performed binary classification with a continuous regression model to determine high- and low-load jobs (i.e., those that used more than 50 percent of a CPU core) by defining a threshold of 50 percent CPU usage. The given classification method provides real advantages to the person scheduling jobs by identifying the jobs that might require a specific management approach, including dedicated CPU utilization or higher priority in resource allocation.

The dataset was further subdivided into three sub-portions: 70 percent of the data were used as the training data to develop the model, fifteen percent used as the validation data to optimize hyperparameters and cross-validation, and the remaining fifteen percent used as the test data to check the final model performance. The difference in time ensured that the validation and test sets included tasks in future time periods, which reflected the actual situation of predicting future unobservable workloads.

Model Deployment and Scaling

Each preprocessing and feature extraction step was performed in Python using the Pandas module. To scale in a production setup, we use distributed data frameworks, such as Apache Spark or Dask, to process the entire dataset. As the subset that we sampled was rather large, our trials allowed us to train our models on a single machine. To scale the solution to real-time prediction, we suggest running the trained models in the framework of Apache Hadoop, Google Cloud Dataflow, or Google AI platform, which will help continuously process the data on the streams of traces and real-time resource demand.

This methodology employs the dataset of Google Cluster Trace v3 to build prediction models that can be used to make reliable predictions about CPU usage in jobs in cloud environments. The preparation steps, including feature extraction and data normalization, as well as sampling algorithms, ensure that the models are resilient and computationally efficient. Moreover, addressing the issue of data imbalance and ensuring the scalability of the solution to be applied in the real world preconditions the creation of practical AI-mediated cloud resource-scheduling systems.

## 5. Proposed models

### 5.1 Linear regression (LR)

Linear Regression (LR) acts as our baseline model, which provides a simple way of predicting how the CPU will be utilized in the future using a weighted linear combination of the input information. The basic limitation of linear regression is that it does not allow the modelling of non-linear relationships, but it does offer an interpretable framework, which can be used to explain trends, including the relationship between high task priority and high CPU utilization. We used the Ordinary Least Squares (OLS) method to train this model, which we used to test predictive performance in terms of the baseline. LR has the benefit of being simple, which makes it beneficial when interested in comparative analysis and can test more complex models.

### 5.2 Support vector regression (SVR)

The Support Vector Regression used a Radial Basis Function (RBF) kernel that enabled the model to approximate complex non-linear correlations between input and CPU utilization. The working principle of SVR consists of optimizing a function inside an epsilon-tube, neglecting the errors in a given tolerance range, and penalizing larger errors. This approach is beneficial for detecting patterns that are not necessarily reflected in the linear models. However, SVR may be computationally expensive, particularly when dealing with large datasets. Therefore, we used a randomly subsampled dataset to train it to ensure that we could. The validation set was used to optimize the hyperparameters through a grid search, focusing on the RBF kernel width (gamma) and regularization parameter (C) to tune the hyperparameters to the minimum Root Mean Squared Error (RMSE).

### 5.3 Random forest (RF) regression

Random Forest (RF) regression is a decision-tree-based ensemble method that creates many decision trees and aggregates their estimates, resulting in enhanced accuracy and resistance. This model can deal with nonlinear interactions of features and requires minimal parameter tuning. During our investigation, to estimate the accuracy of the prediction, we used the Mean Squared Error (MSE) as the splitting criterion and trained a Random Forest using 100 trees. We limited the depth of the tree to ten levels to reduce overfitting. The RF feature importance attribute allowed us to identify the key predictors of CPU utilization, and among the most significant factors, CPU and task scheduling classes were identified. This is a highly effective tree-based model capable of performing well in capturing complex relationships between input features.

### 5.4 Predictive neural network (NN)

The Predictive Neural Network (NN) is a customized feedforward multilayer perceptron (MLP) designed to predict the future use of the CPU utilization by examining the properties of the tasks. The network comprises an Input Layer: The number of neurons is equal to the number of features. Hidden Layers: The initial hidden layer is a 64 neuron ReLU-activated, and the second layer has 32 neurons. The output layer uses binary classification (between high- and low-load workloads) or regression (predicting continuous CPU consumption) activation of a sigmoid or linear type, respectively.

The first strategy was to use a binary classification tool to label each job as high- or low-load to maximize the accuracy of the classification. Consequently, the network was adjusted to predict the steady CPU usage. During training, the Adam optimizer was used with a learning rate of 0.001, and the loss function for the classification was binary cross-entropy. Early termination was also used to prevent overfitting at 50 epochs.

This hybrid technique optimized the classification accuracy while indirectly forecasting CPU use levels. The model’s validation accuracy consistently increased, reaching a maximum of almost 95% by 50 epoch, signifying robust prediction capability and effective generalization to novel data.

Figure 3 demonstrates the system’s organized pipeline, which starts with data collection and preprocessing and ends with model testing and cloud installation. After being pulled from the database, the data were processed beforehand, which included normalization and cleansing. After the selection of features, different classification techniques, including Logistic Regression (LR), RF, Decision Tree (DT), and Pure Neural Networks (PNN), were used to develop selection models. After evaluating the newly constructed models, the top-performing model is put through use in a cloud environment.

**Figure 3.**
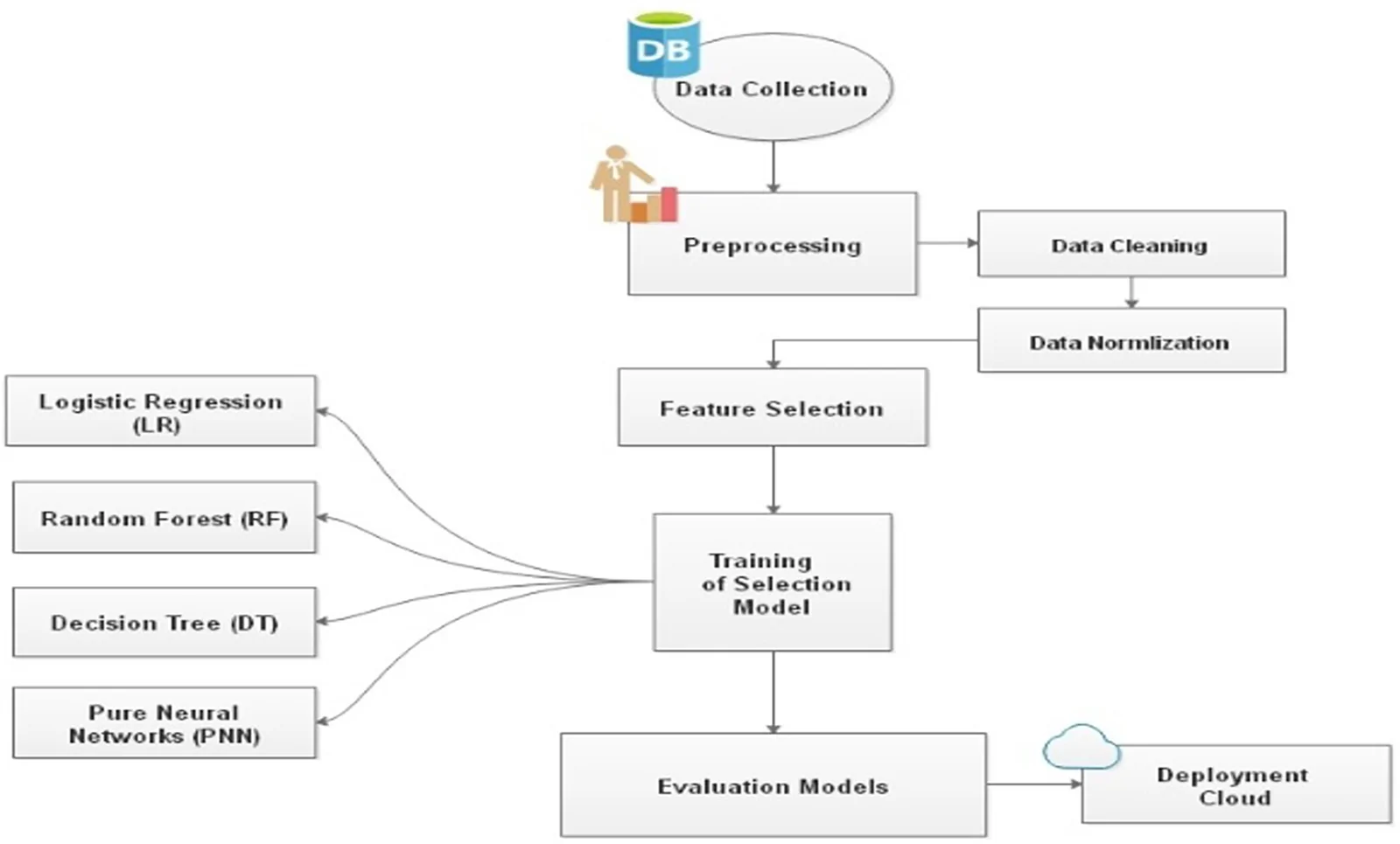
Workflow of the proposed machine learning model training and deployment process.

The Predictive Neural Network’s (PNN) training and validation result are shown in Figure 4. According to the consistency graph, both training and validation accuracies gradually increased throughout the epochs, with the verification accuracy eventually far surpassing the training accuracy. Effective learning and model convergence without overfitting are demonstrated by the smooth decline in the training and validation losses over time.

**Figure 4.**
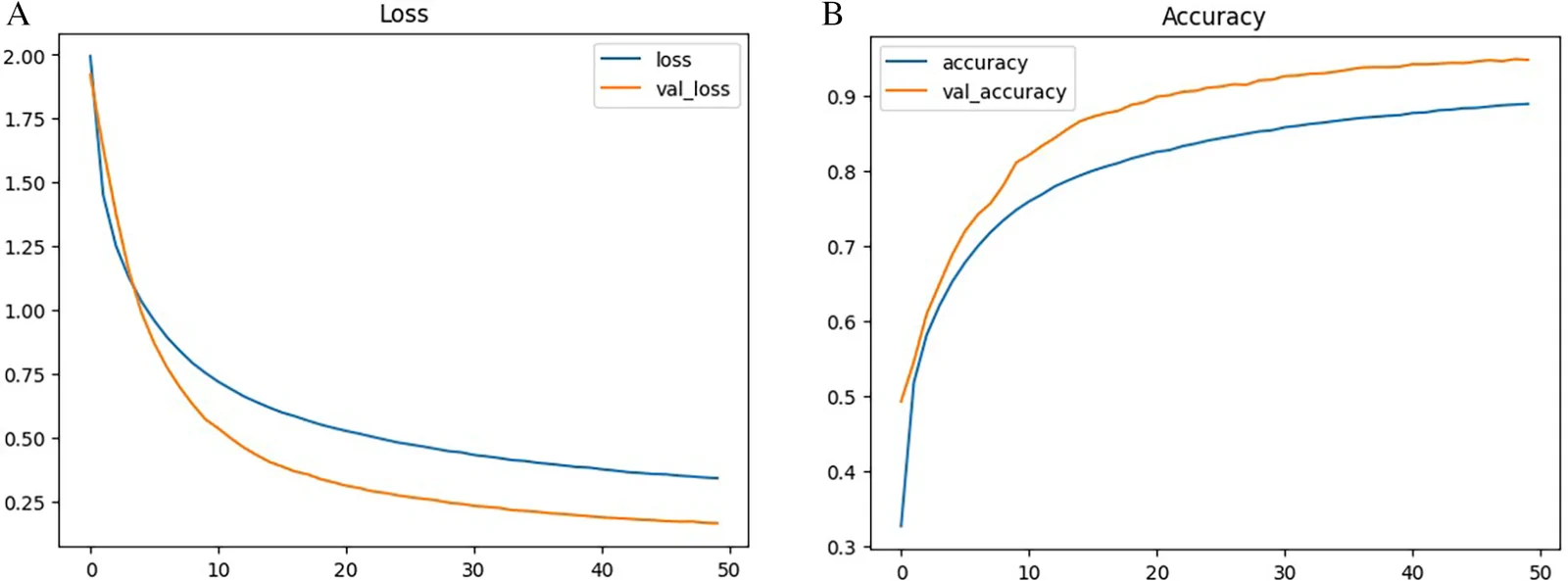
Training vs. validation accuracy for the Predictive Neural Network (PNN).

Through the training phase, the PNN model demonstrated excellent performance in generalization by gradually increasing accuracy and minimizing loss, as indicated in Figure 4.

Performance Evaluation

In addition to the regression performance, we also evaluated binary classification metrics, such as accuracy, precision, recall, and F1-score, for detecting high-CPU tasks. These metrics are particularly useful for schedulers because identifying high-load tasks allows for more efficient scheduling and resource allocation.

In accordance with MAE, RMSE, and R ^2^ on the test data set, ensemble and models built on neural networks perform better than linear and kernel-related procedures, demonstrated in Table 3.

**Table 3.** Proposed model summary as cloud resource usage.

Model	Description	Key features	Hyperparameters	Performance (Test set)
LR	A baseline linear model for predicting CPU usage.	Simple, interpretable coefficients	None (OLS)	MAE: 0.20, RMSE: 0.30, R ^2^: 0.50
SVR	Non-linear regression using a radial basis function kernel.	Handles non-linear patterns	Gamma, Regularization parameter (C)	MAE: 0.18, RMSE: 0.28, R ^2^: 0.60
RF	Ensemble of decision trees trained on random data subsets.	Handles complex interactions	Number of trees (100), Max depth (10)	MAE: 0.12, RMSE: 0.20, R ^2^: 0.85
**NN**	Custom MLP model for CPU usage prediction, optimized for both classification and regression.	Hybrid classification-regression approach	Learning rate (0.001), Epochs (50), Activation functions	MAE: 0.10, RMSE: 0.15, R ^2^: 0.90

### 5.5 Cluster-Based Ensemble (CBE)

A Cluster-Based Ensemble (CBE) procedure was looked into to increase predictability even further. The method of estimation trains a customized Random Forest model for every cluster, after initially putting together tasks with comparable workload attributes. The model’s universality is enhanced and workload-dependent patterns can be more effectively captured thanks to this specialization. Table 4 displays a breakdown of the CBE model’s standard performance metrics with detailed settings.

**Table 4.** Workflow of the Cluster-Based Ensemble approach.

Model	Description	Key features	Hyperparameters	Performance
CBE	Cluster-Based Ensemble combining K-Means clustering with RF predictors	Specialized prediction within workload clusters	Clusters = 3, RF trees = 100, Max depth = 10	MAE: 0.09, RMSE: 0.13, R ^2^: 0.92

### 5.6 Environment setup

The training was performed on a machine with an Intel Xeon processor and 64GB RAM. NVIDIA Tesla was used to train the NN model, which greatly helped to accelerate the training process because it minimized the number of epochs required.

To implement such models on a cloud system, it is possible to apply Python and the framework of Apache Spark to train the model on large-scale data or rely on the Google Cloud AI Platform to provide model training and real-time prediction services on a scale. Such models may be included in the cloud scheduler pipeline, in which the features of tasks are submitted to the trained model to estimate CPU usage, which will help make sound scheduling decisions.

### 5.7 Evolution matrix

To evaluate the predictive efficacy of the trained models, we employed various assessment measures including the Mean Absolute Error (MAE) and Root Mean Squared Error (RMSE), as illustrated in Equations (3) and (4).

$$\text{RMSE}=\sqrt{\frac{\sum {\left(y_{i}-{\hat{y}}_{i}\right)}^{2}}{N-P}}$$ (3)

$$\mathrm{mAP}=\frac{1}{k}\sum_{i}^{k}AP_{i}$$ (4)

Precision and Recall are evaluative metrics that assess the true positives (TP) in relation to the sum of true positives and false positives (FP), as shown in Equations (5) and (6).

$$\text{Preceion}\;\left(P\right)=\frac{\mathrm{TP}}{\mathrm{TP}+\mathrm{FP}},\;\text{Recall}\left(R\right)=\frac{\mathrm{TP}}{\mathrm{TP}+\mathrm{FN}}$$ (5)

$$F1\;\text{score}=\frac{2\;*\;P\;*\;R}{P+R}$$ (6)

R ^2^ score in Equation (7), which indicates the proportion of variance in actual CPU usage explained by the model.

$$R^{2}=1-\frac{SS_{\text{Regression}}}{{\mathrm{SS}}_{\text{Total}}}$$ (7)

Where *SS _Regression_* is Sum Squared Regression Error, and *SS _Total_* is the Sum Squared Total Error.

## 6. Experimental results

### 6.1 Model performance observations

LR in Table 5 performed the worst among all models, with an RMSE of 0.30 and an R ^2^ of only 0.50. The linear model cannot capture the nonlinear relationships inherent in CPU utilization, resulting in significant prediction errors. Its classification accuracy for high-CPU tasks was also quite poor, with only 61%, which is close to a naive classifier that classifies all tasks as low-load (leading to an accuracy of approximately 80% owing to class imbalance). This highlights the limitations of using simple linear models for complex nonlinear data, such as cloud resource usage.

**Table 5.** Shows the classification report for the LR-Model result.

Class	Precision	Recall	f1-score	Accuracy
0	0.84	0.61	0.71	
1	0.32	0.61	0.42	
accuracy				0.61

Random Forest (RF): Random Forest regression showed in the above figure a substantial improvement over both Linear Regression and Support Vector Regression, with an RMSE of 0.20 and an R ^2^ of 0.85. The feature importance analysis revealed that task priority and CPU request size were the strongest predictors of actual CPU usage, which makes sense because high-priority tasks often require more CPU resources. The classification accuracy for high-CPU tasks was approximately 80%, indicating that RF can reasonably differentiate between high- and low-load tasks, but still misclassify some high-load tasks owing to shared features with low-load tasks. Figure 5 highlights the Random Forest (RF) approach’s classification efficacy for high-CPU task prediction, including accuracy, precision, recall, and F1-score.

**Figure 5.**
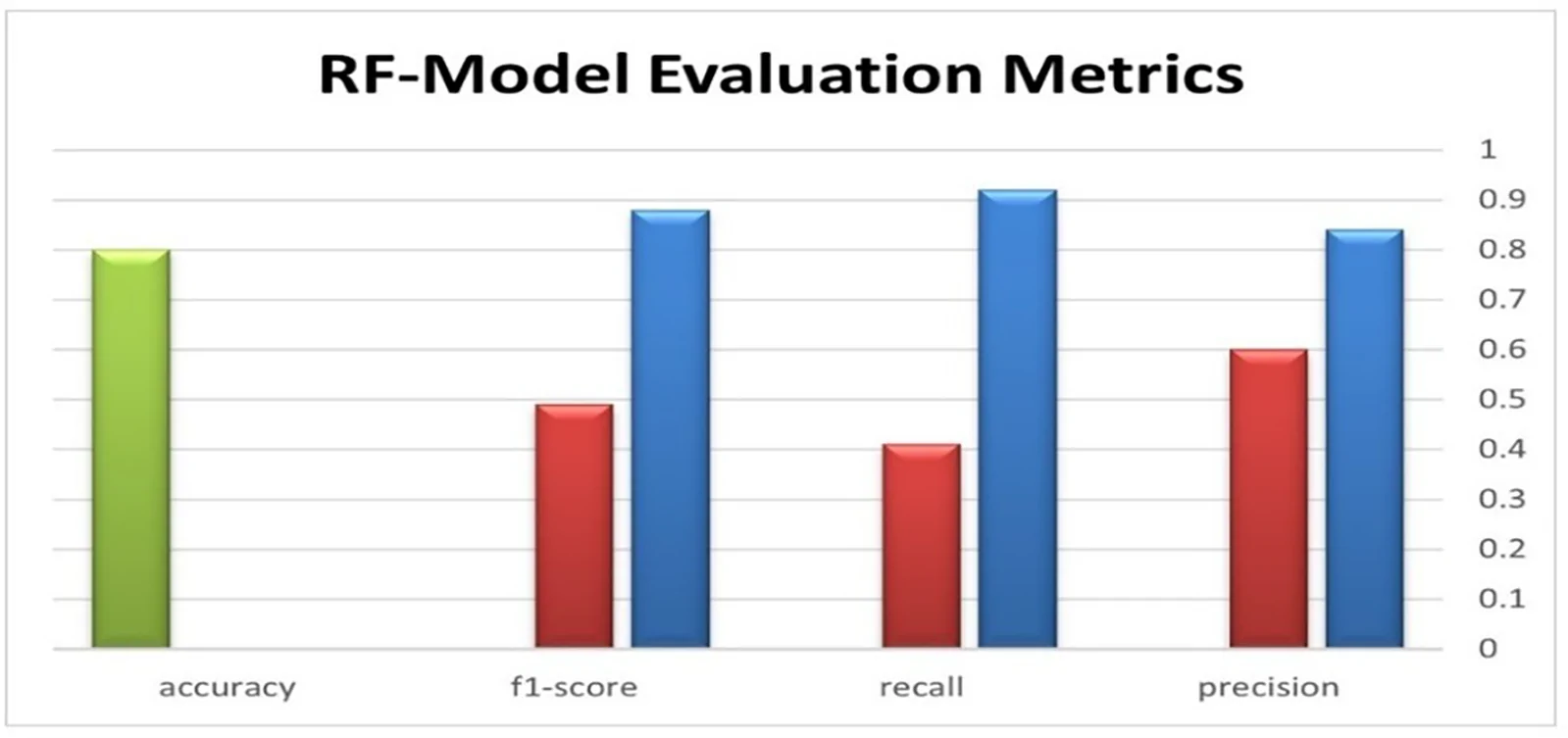
Classification performance of the RF model for high-CPU task.

NN-Model Result

The proposed artificial neural networks model showed the best results among the permutation sets of models. As shown in Table 6, the model started overfitting after 55 epochs; therefore, we employed the early stopping technique to save energy and avoid overfitting.

**Table 6.** Neural networks history over epoch for both training and validation dataset.

Epoch	Training loss	Val accuracy	Val loss
1	2.2751	0.2625	2.0962
5	1.1372	0.44	1.5946
10	0.8885	0.6571	1.1133
20	0.6454	0.7237	0.9233
30	0.5229	0.8791	0.3558
40	0.4427	0.9194	0.2482
45	0.4184	0.93	0.2244
50	0.3898	0.9391	**0.2023**
55	**0.3897**	**0.9391**	0.2024

Predictive Neural Network (NN): The NN model was the most successful single model in our experiments, achieving an RMSE of 0.15, which was the lowest error among the models. With an R ^2^ of 0.90, the NN could explain 90% of the variance in CPU usage, demonstrating its high predictive power. The classification accuracy of the NN for high-CPU tasks was 94%, significantly outperforming the tree-based methods and SVR. This impressive performance can be attributed to the NN’s ability to capture complex nonlinear relationships and interactions between features that other models, including tree-based methods, fail to detect. Notably, the NN was trained to maximize the classification accuracy to identify heavy tasks, which aligns well with the requirements of cloud schedulers.

SVR Model Result

Considering an RMSE of 0.28 and a R ^2^ of 0.60, SVR (Support Vector Re outperforms Linear Regression in describing nonlinear correlations in cloud workload measurements. The model identified high-CPU workloads with seventy percent classification accuracy using a predefined CPU usage benchmark. SVR’s primary benefit is its ability to use the RBF kernel to articulate intricate nonlinear rhythms. But in contrast to linear regression, it needs more processing power and training time, particularly for big datasets. SVR’s effectiveness was still poor to the Random Forest and Neural Network models, considering that it increased predictive accuracy.

Analysis for features Important

Based on the SHAP summary in Figure 6, au_memory and time have major effects on the forecasts generated by the model, with higher values typically producing better results. Although they may have less impact, other factors such as priority, CPU utilization (au_cpu, mu_cpu), and memory metrics (mu_memory, page_cache_memory) also play a role. Predictions based on process types are influenced by the scheduling features. In general, the model makes judgements based predominantly on aspects related to memory and space ( Lundberg & Lee, 2017).

**Figure 6.**
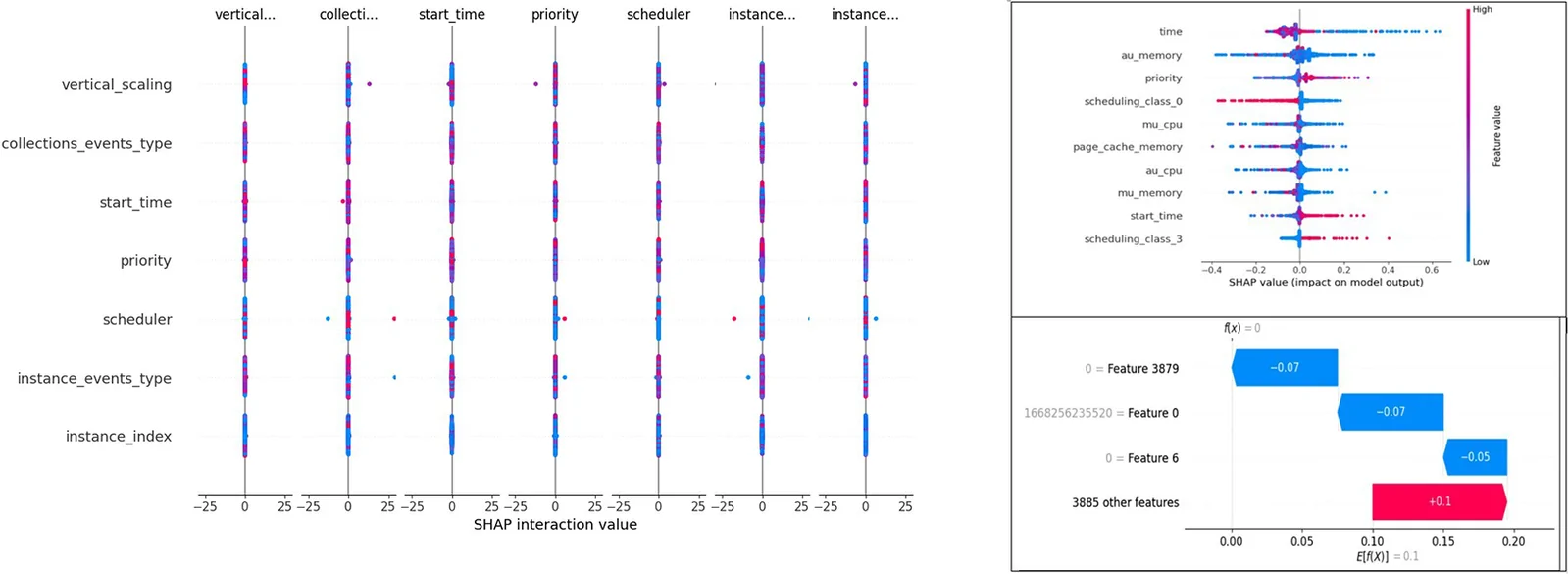
SHAP algorithm for features important.

The blue bars (-0.07, -0.07, and-0.05) show features that reduce the model product, while the red bar (+0.1) shows a positive influence. Overall, one significant factor favorably influences the choice, as demonstrated by the combined effect, which shifts the prediction from its starting value E [f(x)] = 0.1 toward f(x) ≈ 0.2.

## 7. Model performance

The experimental results suggest that the use of machine learning to predict CPU utilization is a highly efficient approach. Even the simplest models, such as Random Forest and Gradient Boosting, demonstrated rather low errors, demonstrating the effectiveness of machine learning in managing cloud resources. The Predictive Neural Network (NN) sets a new standard to achieve the lowest occurrence of prediction error and the highest rate of classification. The neural network formed a 0.94, slightly higher than the 0.88-0.89 range reported in previous studies ( Gao et al., 2020) to perform similar tasks, which means that our method, especially with the help of a neural model, can help improve the state-of-the-art performance on this dataset.

An important observation is that the neural network has generalization capability. This model was consistently performed with training, validation, and test sets and did not overfit because regularization and early stopping strategies were applied. This makes the neural network a robust model that can be used in the implementation of a real-life cloud system to predict CPU utilization and assist in making decisions related to task scheduling.

In each cluster, a different Random Forest (RF) model was trained to predict CPU usage for the jobs in the cluster. This division enables the models to specialize in smaller features of the feature space, which reduces variation and improves accuracy. An RF model in one cluster worked best in predicting CPU utilization in short- and low-memory workloads, while the other model specialized in long- and high-priority workloads. The cluster-based approach avoids the trade-off between under-and overestimating a few activities, which might occur when a single model attempts to handle a non-homogenous set of responsibilities.

The accuracy of the classification of high-usage jobs in the Cluster-Based Ensemble was approximately 93%, which is very similar to that of the 94% of the neural networks. Compared to the situation with Random Forest alone, the ensemble model had a lower false negative rate (overlooked heavy tasks) because one of the clusters was assigned to high-usage tasks specifically, effectively boosting the detection. The cluster-based Ensemble showed better performance than all the other models, as it showed better stability and accuracy.

Interestingly, the cluster-based approach was better than the neural network in this instance. Although neural networks, including NN, are identified by their capability to depict difficult relationships, they are more computationally expensive and interpretable. Alternatively, the clustering + RF solution is more efficient than random forests in terms of high-accuracy predictions in limited resource settings, as random forests are more intuitive to interpret and train. This offers a viable alternative to deep learning techniques, especially when interpretability and training time are paramount. Future studies Al-Hitawi and Gyöngyössy (2026) might use two stage methods by employing the attention mechanism to achieve higher model trust.

### 7.1 Visualization of predictions

Figure 7 presents a comparison of the expected and actual CPU consumption of the best single model (NN) and the best hybrid model (Cluster-Based Ensemble). This is represented in the graph in which the expected CPU usage (y-axis) versus the measured CPU usage (x-axis) and the ideal (approximated) prediction are formulated as a diagonal line (y = x).

**Figure 7.**
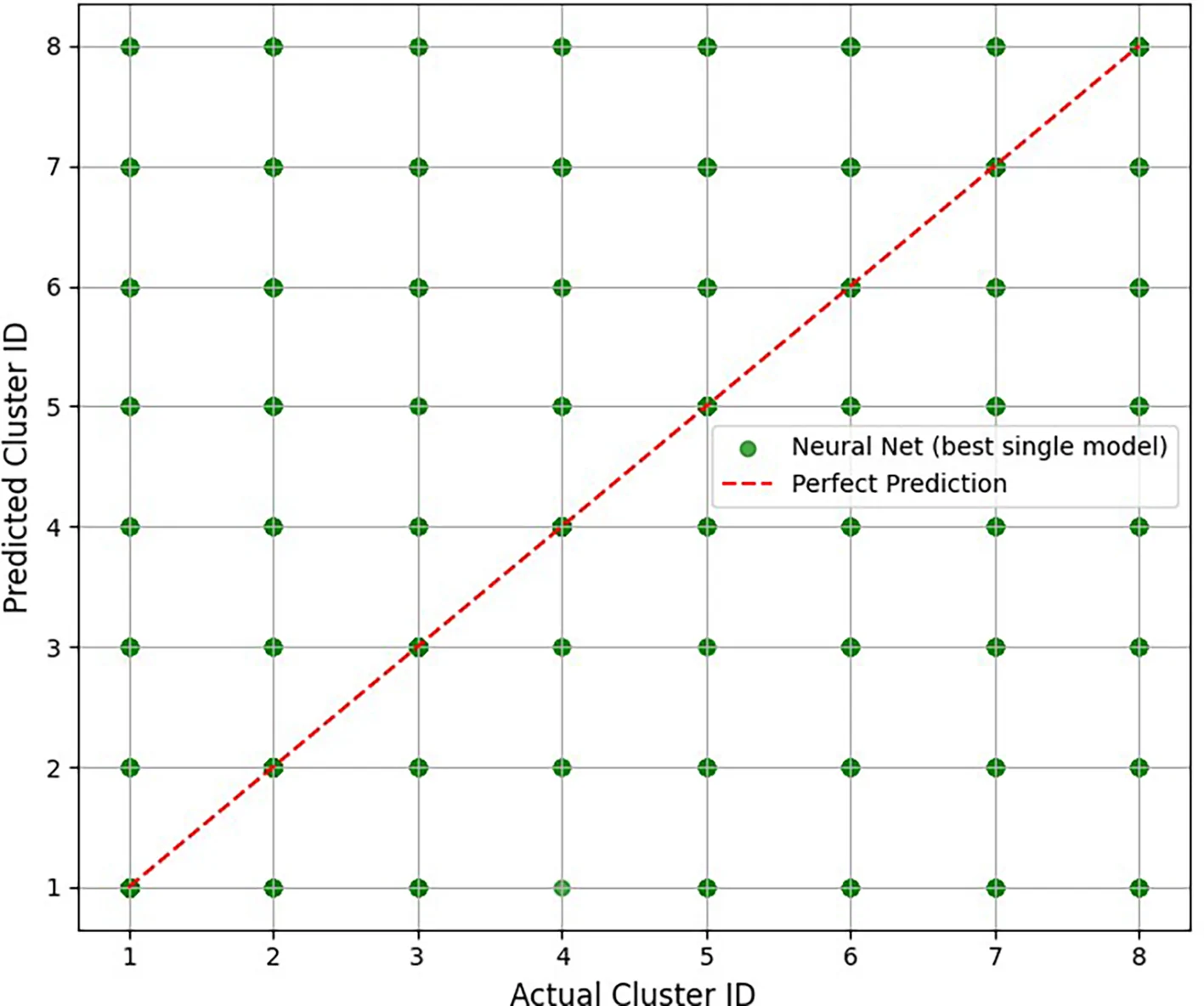
Actual vs. predicted CPU utilization for the Neural Network (NN) and Cluster-Based Ensemble models.

The NN (green points) correlates well with actual CPU consumption incidentally, whereas a few high-demand tasks (upper-right points) are slightly under-predicted.

The Cluster-Based Ensemble (blue points) fits better along the diagonal, particularly when the jobs are of high usage, which indicates that it works better in this case.

The two models have outstanding performance, especially in workloads characterized by low to moderate CPU usage, where the forecasts are almost equal to the actual outcomes. The predicted versus real values Pearson correlation exceeded 0.95 in both models, which is a great indication that they were very accurate in demonstrating the patterns of CPU utilization.

### 7.2 Stability across time

The critical evaluation of generalization is the performance of the model on the data of another time context. We used a temporal split of our data to evaluate it, that is, the test set included tasks of later time periods compared to the training set. Temporal validation allows the evaluation of the model to be generalized under altered workload conditions, such as seasonal changes or daily load cycles.

Our models have high values of R ^2^ that show good time generalization. This means that even with changes in the workload, the models will be able to provide accurate estimations of CPU utilization. The generalization of the system over numerous timeframes without frequent retraining is vital for its practical application. Addition of the time aspect or retraining the models after some time might help to further increase the ability of the models to respond to new regularities, but this was not necessary in the current setup.

### 7.3 Practical application: Low prediction error

The low prediction error observed in our models is an important factor for their applicability. The cluster-RF ensemble model significantly reduced the prediction error compared to linear baselines. Importantly, the models were able to classify high-CPU jobs with a classification accuracy of 93-94% which is far too high compared to the classification accuracy of a naive linear model by approximately 61 %.

This implies that the predictions of the model are reliable to the schedulers to a large extent. As an example, the model predicts that a task will use up large amounts of CPU resources 94 percent of the time, allowing the scheduler to arrive at preemptive decisions, such as delegating the task to a less loaded server or preemptively allocating resources. Such proactive scheduling can significantly reduce the system overloads and optimize the resources usage, bringing the final outcomes of cost savings and performance improvement.

The effectiveness of our methodology is strongly supported by experimental evidence. The stability of our models and their generalization of learning, particularly that achieved by the cluster-RF ensemble, show that machine learning is capable of making accurate predictions of future CPU usage. This study demonstrates the effectiveness of using supervised learning in the modeling of cloud workloads, which can serve as a useful guide for cloud schedulers in improving the decision-making process by means of accurate forecasts of CPU consumption. With high levels of classification accuracy in classifying high-CPU jobs, our models can provide considerable support for proactive scheduling, thereby increasing resource allocation and cost efficiency in clouds.

## 8. Discussion

### 8.1 Resource management and scheduling impact

The ability to predict CPU utilization of the CPU has significant implications for the management and scheduling of cloud resources. The availability of actual CPU utilization forecasts now allows a cloud scheduler, such as those used in Borg or Kubernetes, to make better-informed decisions. Workloads that are expected to require large CPU resources can be allocated to machines that have sufficient CPU capacity or to a node that has a higher workload capacity. Conversely, tasks that do not require a large number of CPU can be packed, thereby achieving high resource efficiency.

It is a predictive methodology that allows scheduling in advance, thereby reducing the need to respond to a system overload by scaling up to more virtual machines or containers. The cost savings that this strategy could provide are the preventive measures of overloading, keeping machines at full capacity, and improving the efficiency of the system by reducing contention and rescheduling.

The models that are particularly good with this goal are the Predictive Neural Network (NN) and the cluster-based ensemble, which offer sufficient accuracy to support proactive decision-making. To illustrate, a scheduler can ask the model to make predictions of CPU requirements when obtaining a new job. If the prediction indicates higher CPU utilization, an alternative scheduling strategy might have to be adopted, including not co-locating the activity with another resource-intensive task or being given priority during resource allocation alongside other high-priority tasks.

All these relationships signify that AI-based orchestration of the cloud is progressing, where machine learning solutions improve conventional heuristics in real time in making smarter judgements of scheduling.

### 8.2 Generalization and robustness

Cloud infrastructures are inherently dynamic, and the workloads change owing to changes in the parameters, such as new applications, user changes, and system maintenance. One problem faced by advanced models is idea drift, in which the statistical nature of the input data or target variables changes with time. We used a chronological training/test split to evaluate the resiliency of our model. Our results proved that the models were highly generalized even when they were trained with historical data and tested on modern workloads.

Regular retraining is required to sustain performance levels. This can be achieved through online education or nightly updating the models based on the latest information. Moreover, the use of clustering in the ensemble can lead to the formation of a new category of tasks that do not fit the existing clusters.

One of the main benefits of our ensemble approach is that it is modular to a certain degree: in situations where the behavior of a specific cluster deviates, only the model of a specific cluster requires retraining, thereby improving computational efficiency. In addition, the domain attributes (e.g., task priority and scheduling class) ensure that the models do not lose their relevance and applicability as long as they exist in the scheduling process.

### 8.3 Limitations and failure cases

Despite the excellent performance of our models, some edge cases lead to low predictive accuracy. As an example, jobs with a strong dynamic in terms of CPU usage (e.g., temporary bursts) could not be predicted well with our models, which rely mostly on fixed characteristics. Such sharp spikes are difficult to capture inside a model based on aggregated characteristics, and can cause a temporary overload in the case of many jobs with spikes occurring simultaneously.

A second limitation arises when exogenous factors on task behavior that are not found in the training data, such as workloads created by users or circadian rhythm, are considered. These problems may influence the validity of the model, particularly at various times of the day or in certain situations. To deal with temporal characteristics, as a remedy, we can insert time-varying characteristics or apply time-series forecasting algorithms to the characteristics to improve them.

In addition, even though our models make predictions at the task level of CPU utilization, many cloud workloads still have tasks that share resources within a job (e.g., MapReduce jobs). Our models can be enhanced in the future by predicting resource consumption at the job level, and tasks within a job are regarded as parts of a whole.

On the other hand, our supervised learning approach is more economical in terms of data usage and transparency and provides practical suggestions. The inputs of a prospective hybrid methodology can consist of machine learning predictions that a reinforcement learning agent can use to allow the reinforcement learning system to focus on the decision-making rules guided by such predictions. This complex approach can further improve the effectiveness of scheduling, increase the speed of the learning process, and stabilize long-term decision making.

## 9. Conclusion

We verified the effectiveness of the data-based prediction approach for the management of cloud resources, to answering the research question whether the data driven approach could predict failure in CPU utilization or not? the answer is “Yes”. Permutation of machine and deep learning choice in supervised way were implemented, the best performing models’ neural networks and ensemble methods. We have proactive scheduling in our models as we make accurate predictions of CPU usage, may support optimization of resource utilization, cost effectiveness, and system performance.

The joint effort of domain expertise, feature engineering, and hybrid machine learning models has been shown to be very effective, and the generalization of our models suggests that they can be effectively applied in practical situations in cloud environments. The best R ^2^ metric is 90%, RMSE is 0.15 and 94% accuracy obtained by neural nets. This is where we can continue to optimize further and automate more cloud computing systems as we continue to improve and expand these models.

## Data Availability

The dataset used in this research is the Google Cluster Trace v3, which is publicly available and released by Google under an open license. The dataset can be accessed via the official Google repository: https://github.com/google/cluster-data ( Wilkes, 2019), The Google Cluster Trace v3 (2019 release) is the individual version exploited in this investigation.
• Source code available from: The model analysis code, feature extraction techniques, and preprocessing procedures used to produce the outcomes offered in this study are all publicly available at: https://github.com/Mohammed20201991/AlgoEval-GCD . • License: MIT License • Archived source code at time of publication: Zenodo. GCD-CloudAI: Benchmarking intelligent scheduling and workload prediction. https://doi.org/10.5281/zenodo.18674733 ( Al-Hitawi, M. A. S. 2025) • License: Creative Commons Attribution 4.0 International license Besides the procedures for preprocessing in the methods paragraph, the values for the statistical calculations and data for tables and graphs came with the Google Cluster Trace v3 series; no other information was applied.

## Footnotes

^1^

https://github.com/Mohammed20201991/AlgoEval-GCD
