# Supplementary material for: Intelligent Cloud Resource Usage Potentially to Improve Task Scheduling by the use of Artificial Intelligence
Source: F1000Res. 2026 Sep 30;15:366. Originally published 2026 Mar 5. [Version 3] doi: 10.12688/f1000research.177203.3 (PMC13643142; doi:10.12688/f1000research.177203.3)
Supplement: Peer review history

# Reviewer report: Reviewer response for version 3

**DOI:** 10.5256/f1000research.209626.r525683

**Published:** 2026-10-06

**Authors:** Alexandra Theodoropoulou

**Affiliations:** 1 University of Patras, Patras, Greece

The authors have mostly addressed my suggested revisions, therefore I'd say the manuscript can now be indexed.

Is the work clearly and accurately presented and does it cite the current literature?

No

If applicable, is the statistical analysis and its interpretation appropriate?

Partly

Are all the source data underlying the results available to ensure full reproducibility?

Partly

Is the study design appropriate and is the work technically sound?

No

Are the conclusions drawn adequately supported by the results?

No

Are sufficient details of methods and analysis provided to allow replication by others?

Partly

Reviewer Expertise:

Big Data analytics, Machine Learning, Decision Support Systems, Data-driven Management, Digital Marketing, Consumer Behavior, Business Intelligence, Applied Artificial Intelligence

I confirm that I have read this submission and believe that I have an appropriate level of expertise to confirm that it is of an acceptable scientific standard.

# Reviewer report: Reviewer response for version 2

**DOI:** 10.5256/f1000research.203354.r496558

**Published:** 2026-06-29

**Authors:** Alexandra Theodoropoulou

**Affiliations:** 1 University of Patras, Patras, Greece

The authors have addressed some of my revision comments, particularly by revising parts of the Abstract, expanding the literature discussion, adding some clarification to the methodology, and improving parts of the results presentation. However, the revised manuscript still contains several unresolved issues that require further correction. More specifically, the target-variable definition, the consistency of the model set, the presentation of the experimental results, the claimed classification-accuracy optimization, and the strength of the practical conclusions remain insufficiently addressed within the manuscript. Therefore, although the first revision has shown some progress, the manuscript still needs further changes before it can be considered satisfactory.

 Suggested Revisions

  

1. The target variable is still not defined clearly enough. The manuscript says that the model predicts future CPU usage using scheduling-time information, but it also defines CPU usage through execution-time quantities. The authors should define the target variable once and clearly state which features are available before execution and which, if any, are only known after execution.
2. The results presentation remains inconsistent across models. The manuscript discusses LR, SVR, RF, NN, and CBE but Figure 3 still includes only Logistic Regression and Decision Tree, which are not consistently developed in the methods. The authors should make the model list, model names, figures, and results tables fully consistent.
3. The claim that the neural network was optimized for classification accuracy is still not sufficiently supported. The manuscript reports increasing validation accuracy, but this does not demonstrate an explicit optimization procedure. The authors should either explain the actual optimization process, including threshold tuning or model selection, or remove this claim.
4. The conclusion still overstates the practical implications of the experiments. The study reports prediction metrics, but it does not evaluate scheduler-level performance, cost reduction, overload reduction, or actual resource-allocation improvement. The authors should tone down the conclusion and state that the results show predictive potential, not proven operational efficiency.

Is the work clearly and accurately presented and does it cite the current literature?

No

If applicable, is the statistical analysis and its interpretation appropriate?

Partly

Are all the source data underlying the results available to ensure full reproducibility?

Partly

Is the study design appropriate and is the work technically sound?

No

Are the conclusions drawn adequately supported by the results?

No

Are sufficient details of methods and analysis provided to allow replication by others?

Partly

Reviewer Expertise:

Big Data analytics, Machine Learning, Decision Support Systems, Data-driven Management, Digital Marketing, Consumer Behavior, Business Intelligence, Applied Artificial Intelligence

I confirm that I have read this submission and believe that I have an appropriate level of expertise to state that I do not consider it to be of an acceptable scientific standard, for reasons outlined above.

# Response

**Published:** 2026-08-11

**Authors:** Mohammed Abduljawad Al-Shibly

We sincerely thank the reviewer for the careful assessment and constructive comments. The manuscript has been further revised to address the remaining concerns. The detailed revisions are incorporated in the updated manuscript attached to the email submitted to the Editorial Board, as summarized below:

 1. Target variable definition

 The target variable has been clearly redefined as future CPU utilization, with prediction inputs restricted to information available at or before task dispatch. Post-execution variables were excluded to avoid information leakage.

 2. Consistency of models and results

 The model set, figures, tables, and terminology have been revised for consistency. Logistic Regression and Decision Tree have been removed, and the evaluated models are now consistently presented as Linear Regression (LR), Support Vector Regression (SVR), Random Forest (RF), Neural Network (NN), and Cluster-Based Ensemble (CBE).

 3. Neural-network classification optimization

 The unsupported claim regarding optimization for classification accuracy has been removed. The NN is now presented primarily as a regression model, while high-/low-CPU classification is treated as a secondary analysis based on continuous CPU-utilization predictions.

 4. Practical implications and conclusion

 The conclusion has been revised to avoid overstating the practical implications. The findings are now presented as demonstrating the predictive potential of the proposed models rather than proven improvements in scheduling efficiency, cost reduction, overload reduction, or resource allocation. Scheduler-level evaluation is identified as future work.

 We appreciate the reviewer’s constructive comments, which have helped us improve the clarity, methodological consistency, and scientific interpretation of the manuscript. The corresponding changes can be found in the updated manuscript attached to the email submitted to the Editorial Board.

# Reviewer report: Reviewer response for version 1

**DOI:** 10.5256/f1000research.195388.r467290

**Published:** 2026-05-21

**Authors:** Chedi Morchdi

**Affiliations:** 1 Texas A&M University, Texas, USA

This article presents a relevant and well-executed study on cloud CPU usage prediction using the Google Cluster Trace v3 dataset. The manuscript clearly explains the practical motivation—improving task scheduling and resource allocation under highly variable cloud workloads—and evaluates several supervised models, including Linear Regression, Support Vector Regression, Random Forest, and Neural Networks. The reported results are strong, with the neural-network model achieving about **94% validation accuracy** and **R² = 0.90**, which supports the paper’s main contribution.

 The study design is appropriate and technically sound. The dataset contains rich task-level information such as start and end times, CPU and memory usage, scheduling class, and priority, which are relevant predictors for the target problem. The paper also includes preprocessing, feature extraction, comparative model evaluation, and discussion of hybrid and ensemble directions, making the analysis coherent and useful for the cloud-computing community.

 The manuscript also supports reproducibility well by using a **publicly available dataset**, and by providing both **source code** and an **archived Zenodo record** for the implementation and preprocessing pipeline. The conclusions are consistent with the experimental findings and are reasonably linked to practical scheduling improvements in cloud environments. Overall, this is a solid and valuable contribution.

Is the work clearly and accurately presented and does it cite the current literature?

Yes

If applicable, is the statistical analysis and its interpretation appropriate?

Yes

Are all the source data underlying the results available to ensure full reproducibility?

Yes

Is the study design appropriate and is the work technically sound?

Yes

Are the conclusions drawn adequately supported by the results?

Yes

Are sufficient details of methods and analysis provided to allow replication by others?

Yes

Reviewer Expertise:

Reinforcement learning, Deep learning, Machine learning, task graph scheduling

I confirm that I have read this submission and believe that I have an appropriate level of expertise to confirm that it is of an acceptable scientific standard.

# Reviewer report: Reviewer response for version 1

**DOI:** 10.5256/f1000research.195388.r469599

**Published:** 2026-04-01

**Authors:** Alexandra Theodoropoulou

**Affiliations:** 1 University of Patras, Patras, Greece

**Abstract**

 The Abstract is a little inconsistent with the main body of the manuscript. In the Abstract, the authors initially state that the evaluated regression algorithms were Linear Regression, Support Vector Regression, and Random Forest, yet they then report neural network performance as a key result. Since the manuscript later includes a dedicated neural-network model section and reports NN results in the main results table, the Abstract should be revised so that the list of evaluated models is complete and the phrasing used is consistent.

**Dataset preprocessing and Feature engineering**

 In section 3 and subsection 4.1 the authors describe the dataset’s features in the exact same way, repeating themselves (“The dataset comprises several critical……assigned by the scheduler)”). Therefore, the authors need to choose one section and describe their dataset there, once. No need to repeat this in other subsections.

 The exact same thing is repeated a little later in subsection 4.2, where the authors repeat the same description about binary classification, first in “Defining the Target Variable” and then in “Dealing with Imbalanced Data” portions of 4.2. This pattern goes on for the 2nd paragraph of “Dealing with Imbalanced Data” as well, which is first mentioned in the “Data Volume and Sampling” part of the paper, but with a few words changed (but the method and approach are the same). Again, the authors need to choose where these parts fit their paper best and keep these descriptions only once throughout the paper.

**Target variable**

 The manuscript is not consistent in how the target variable is defined. In one part of the methods, the study is described as predicting future CPU usage during an upcoming scheduling interval using information available at task dispatch. However, later the dependent variable is defined as CPU utilization during execution, computed from realized execution-time quantities. These are not the same prediction tasks. This inconsistency is not minor because it is unclear whether the model is truly forecasting future CPU demand at scheduling time or whether it is using information that would only be known after execution has already taken place. The authors need to define the target variable once, clearly and consistently, and explicitly state which input features are available at prediction time. If any post-execution information is used, that creates leakage and would make the reported scheduling-oriented claims much less reliable.

**Experimental Results**

 The results are not presented consistently across models, which makes the comparison incomplete and uneven. Linear Regression is given a classification table, Random Forest is presented through a figure that does not clearly report exact values and lacks proper legend clarity, and the neural network is discussed through a training-history table. Support Vector Regression, although described in the methods and included in the summary table, has no dedicated results presentation beyond a single row of metrics. In addition, Figure 3 introduces a different set of models than the main methods section, replacing Support Vector Regression with Decision Tree, which is not otherwise developed or evaluated in the study. The same figure also refers to Logistic Regression, whereas the methods and model description refer to Linear Regression. These are not the same model: Linear Regression is used for continuous prediction, while Logistic Regression is a classification model. The manuscript should therefore be corrected so that the model set, model names, and results presentation are accurate and consistent throughout, and the authors should clearly state which models were actually used and how each one was evaluated.

**Classification accuracy optimization**

 The manuscript states that the neural network was trained to optimize classification accuracy for identifying high-CPU tasks. However, the paper does not show a clear optimization procedure supporting this claim anywhere. It does report increasing validation accuracy during training, but this is not the same as demonstrating that classification accuracy was explicitly optimized through either model selection and threshold tuning, or comparative experimentation. This issue is made less clear by the fact that the NN is also presented as a hybrid classification-regression model, while the final results are reported mainly with regression metrics.

**Reporting**

 There are still contradictions in the way the dataset size and evaluation splits are reported. In the methodology, in subsection 4.2 (Data Volume and Sampling), the sampled subset is described as “tens of thousands of task instances”, but Table 2 reports 405,894 observations, which is much larger and should be stated consistently throughout the paper. There is also inconsistency in how the evaluation split is reported. In addition, the text states that model comparison in Table 3 is based on MAE, RMSE, and R2 on the Test dataset, whereas the performance column in Table 3 is labeled “Validation set”. The authors should therefore report the exact final sample size consistently and make clear which dataset partition each reported metric refers to.

**Figure 7**

 In Section 7.1, Figure 7 is described as showing actual versus predicted CPU consumption for the best single model and the best hybrid model, with predicted CPU usage on one axis and measured CPU usage on the other. However, the figure caption states ‘Actual vs. predicted value grouped by cluster name (node name),’ which does not match that description. The authors need to clarify exactly what Figure 7 shows and make the caption, axes, and in-text explanation fully consistent.

**Conclusion**

 The conclusion overstates what the experiments actually show. The manuscript claims that the results prove machine learning is a highly efficient approach for CPU prediction and cloud resource management. However, the study only reports predictive results such as accuracy, R², and error values. It does not show scheduler-level experiments, comparison with an actual scheduling baseline, or direct evidence of practical gains such as lower cost, reduced overload, or better task placement. In its current form, the paper supports the claim that some models predict CPU usage reasonably well on this dataset, but not the stronger claim that the approach is already proven to be highly efficient in practice. The authors should either tone down this conclusion or add direct evidence connecting prediction performance to actual scheduling improvement.

**References used in the paper**

 The references used are too few for the literature part of the paper, thus undermining the background claims by the authors. The authors should add more relevant work to support their background claims in Introduction and Related Work sections (especially subsection 2.4).

 Overall, I cannot recommend approval of the manuscript in its current form. The paper contains too many substantial inconsistencies between the stated methodology, the reported models, the target definition, the evaluation setup and the presented results. These issues go beyond presentation quality and raise concerns about the reliability, clarity and reproducibility of the study.

Is the work clearly and accurately presented and does it cite the current literature?

No

If applicable, is the statistical analysis and its interpretation appropriate?

Partly

Are all the source data underlying the results available to ensure full reproducibility?

Partly

Is the study design appropriate and is the work technically sound?

No

Are the conclusions drawn adequately supported by the results?

No

Are sufficient details of methods and analysis provided to allow replication by others?

Partly

Reviewer Expertise:

Big Data analytics, Machine Learning, Decision Support Systems, Data-driven Management, Digital Marketing, Consumer Behavior, Business Intelligence, Applied Artificial Intelligence

I confirm that I have read this submission and believe that I have an appropriate level of expertise to state that I do not consider it to be of an acceptable scientific standard, for reasons outlined above.

# Response

**Published:** 2026-05-20

**Authors:** Mohammed Abduljawad Al-Shibly

We sincerely thank the reviewer for the detailed, constructive, and technically insightful evaluation of our manuscript.

 We highly appreciate the reviewer’s careful reading of the manuscript and the valuable suggestions provided. We have carefully revised the manuscript to address all concerns raised in the review. The revised Version 2 includes substantial improvements in methodological clarity, consistency of terminology, experimental reporting, target-variable definition, figure descriptions, and literature coverage.

 Point-by-point response to all comments.

 - Comment: Abstract

 The Abstract is a little inconsistent with the main body of the manuscript. In the Abstract, the authors initially state that the evaluated regression algorithms were Linear Regression, Support Vector Regression, and Random Forest, yet they then report neural network performance as a key result. Since the manuscript later includes a dedicated neural-network model section and reports NN results in the main results table, the Abstract should be revised so that the list of evaluated models is complete and the phrasing used is consistent.

 Response

 We thank the reviewer for identifying this inconsistency. We agree that the original abstract incompletely described the evaluated models and did not fully align with the methodology and results sections. The Changes in Version 2

 The Abstract has been revised to consistently include all evaluated models:

 - Linear Regression (LR),

 - Support Vector Regression (SVR),

 - Random Forest (RF),

 - Predictive Neural Network (NN).

 - The wording of the results summary has been updated for consistency with the experimental sections.

 - The revised abstract now clearly states that the neural network model achieved the best predictive performance among all evaluated methods.

 --------------------------------------

 - Comment: In section 3 and subsection 4.1 the authors describe the dataset’s features in the exact same way, repeating themselves...

 Response

 We appreciate this observation. We agree that several descriptions of dataset attributes and preprocessing procedures were unnecessarily repeated across Sections 3 and 4.

 - Redundant descriptions of dataset features were removed from subsection 4.1.

 - The complete dataset description is now presented only once in Section 3.

 - Repeated explanations regarding binary classification and data splitting in subsection 4.2 were consolidated and rewritten for clarity.

 - The “Defining the Target Variable,” “Dealing with Imbalanced Data,” and “Data Volume and Sampling” subsections were reorganized to avoid duplication and improve readability.

 --------------------------------------

 - Comment: Target variable, The manuscript is not consistent in how the target variable is defined ... to improves the methodological transparency and reproducibility 

 Response

 We agree that the original manuscript did not clearly distinguish between the prediction target and runtime measurements used for evaluation. We also acknowledge that the wording may have unintentionally created ambiguity regarding the availability of features at prediction time.

 We would like to clarify that the predictive models were designed to estimate future CPU utilization using only information available at task scheduling/dispatch time. No post-execution information was used as input features during model training or inference.

 The target variable definition was completely rewritten for consistency throughout the manuscript. We explicitly clarified that:

 - The prediction target is future CPU utilization,

 - Only scheduling-time features were used as model inputs.

 - A dedicated paragraph was added specifying which features are available at prediction time.

 - Ambiguous wording related to execution-time quantities was removed or revised.

 - Additional clarification was added to explicitly state that no data leakage from post-execution variables was introduced into the predictive models.

 -----------------------------------------

 - Comment: Experimental Results, The results are not presented consistently across models. 

 Response

 We agree that the original manuscript contained inconsistencies in model naming, figure terminology, and result presentation formats.

 Changes in Version 2

 The terminology for all models has been standardized throughout the manuscript.

 References to “Logistic Regression” in Figure 3 were corrected to “Linear Regression.”

 The unintended “Decision Tree” reference in Figure 3 was removed because it was not part of the evaluated model set.

 Figure 3 was redesigned to accurately reflect the implemented methodology.

 Additional quantitative performance details for SVR were added.

 A unified comparative results table was introduced to present:

 MAE,

 RMSE,

 R²,

 Accuracy,

 Precision,

 Recall,

 F1-score

 consistently across all evaluated models.

 Figure legends and captions were improved for clarity and consistency.

 --------------------------------------------

 - Comment: Classification accuracy optimization: The manuscript states that the neural network was trained to optimize classification accuracy.

 Response

 We agree that the original wording overstated the optimization process and did not clearly explain the relationship between the regression and classification components of the neural-network model.

 The description of the neural-network training process was revised for accuracy.

 We clarified that: the primary task was regression-based CPU utilization prediction,

 classification metrics were additionally evaluated by applying a threshold to predicted CPU usage values.

 Statements implying explicit optimization of classification accuracy were revised to avoid overstatement.

 The relationship between regression outputs and derived classification metrics is now clearly explained.

 ----------------------------------------------

 - Comment:  Reporting, There are still contradictions in the way the dataset size and evaluation splits are reported...

 Response

 We appreciate the reviewer’s attention to reporting consistency. We agree that the dataset size and evaluation partition descriptions were not consistently stated in the original version.

 The exact final dataset size (405,894 observations) is now consistently reported throughout the manuscript.

 The phrase “tens of thousands” was removed to avoid inconsistency.

 The dataset partitioning strategy was clarified and consistently reported:

 70% training,

 15% validation,

 15% testing.

 Table 3 was revised to explicitly identify whether metrics correspond to validation or test datasets.

 Additional clarification was added regarding temporal splitting methodology.

 -----------------------------------------------------------------------------

 - Comment: In Section 7.1, Figure 7 is described as showing actual versus predicted CPU consumption.

 Response

 Figure 7 caption was rewritten for consistency with the manuscript discussion.

 Axis labels were clarified.

 The in-text explanation was revised to explicitly describe:

 actual CPU utilization,

 predicted CPU utilization,

 and the comparison between the NN and cluster-based ensemble models.

 The misleading wording related to “cluster name (node name)” was corrected.

 -----------------------------------------------------------------------------

 - Comment: The conclusion overstates what the experiments actually show.

 Response

 The conclusion section was carefully revised to avoid overstating practical scheduling impact.

 Claims regarding resource optimization and scheduling efficiency were moderated.

 The revised conclusion now states that the proposed models demonstrate strong predictive capability and potential usefulness for proactive scheduling assistance, rather than claiming proven operational deployment benefits.

 Additional wording was added to clarify that scheduler-level integration and operational evaluation remain important future work directions.

 -----------------------------------------------------------------------------

 - Comment: The references used are too few for the literature part of the paper..

 Response

 We agree that the literature review can be strengthened by incorporating additional recent and relevant studies.

 The Introduction and Related Work sections were substantially expanded.

 Multiple recent references related to:

 cloud workload prediction,

 AI-driven scheduling,

 ensemble learning,

 deep learning for cloud resource management,

 and predictive cloud analytics

 were added.

 Subsection 2.4 was revised and expanded to better position the contribution of this study within current research trends.

 ---------------------------------------------

 Finally

 We sincerely thank the reviewer again for the detailed and constructive comments. The reviewer’s feedback significantly helped improve the quality, clarity, consistency, and reproducibility of the manuscript.

 The revised Version 2 includes:

 - Clearer methodological explanations,

 - Improved consistency in terminology and reporting,

 - Corrected figures and tables,

 - Refined conclusions,

 - Additional literature coverage, and improved experimental presentation.

 We believe that these revisions substantially strengthen the manuscript and address the concerns raised in the review.
